# Internal models in active self-motion estimation: role of inertial sensory cues

**DOI:** 10.1152/jn.00281.2024

**Published:** 2025-06-14

**Authors:** Milou J.L. van Helvert, Luc P.J. Selen, Robert J. van Beers, W. Pieter Medendorp

**Affiliations:** 1 Radboud University, Donders Institute for Brain, Cognition and Behaviour, Nijmegen, The Netherlands; 2 Vrije Universiteit Amsterdam, Department of Human Movement Sciences, Amsterdam, The Netherlands

**Keywords:** Vestibular system, prediction, closed-loop, internal model, self-motion, active

## Abstract

Self-motion estimation is thought to depend on sensory information as well as on sensory predictions derived from motor output. In driving, the inertial motion cues (vestibular and somatosensory cues) can in principle be predicted based on the steering motor commands if an accurate internal model of the steering dynamics is available. Here, we used a closed-loop steering experiment to examine whether participants can build such an internal model of the steering dynamics. Participants steered a motion platform on which they were seated to align their body with a memorized visual target in complete darkness. We varied the gain between the steering wheel angle and the velocity of the motion platform across trials in three different ways: unpredictable (white noise), moderately predictable (random walk), or highly predictable (constant gain). We examined whether participants took the across-trial predictability of the gain into account to control their steering (internal model hypothesis), or whether they simply integrated the inertial feedback over time to estimate their travelled distance (path integration hypothesis). Results show that participants relied on the gain of the previous trial more when it followed a random walk across trials than when it varied unpredictably across trials. Furthermore, on interleaved trials with a large jump in the gain, participants made fast corrective responses, irrespective of gain predictability, showing they also relied on inertial feedback next to predictions. These findings suggest that the brain can construct an internal model of the steering dynamics to predict the inertial sensory consequences in driving and self-motion estimation.

## Introduction

Sensory feedback, especially visual and inertial motion cues, is important in self-motion estimation. Inertial motion information is transduced directly by the vestibular organs, particularly the otoliths, but also by somatosensory cues, such as tactile and pressure cues on the body. People can estimate their self-motion from visual (1) or inertial (2) information alone, but are more precise when feedback from both is available and integrated (3–7).

When the motion is generated actively, self-motion estimates also depend on predictions from internal models of sensory and body dynamics that transform motor commands into predicted sensory consequences (8,9). In combination with actual sensory feedback, these predictions lead to better estimates of self-motion (10–14), also in patients with vestibular deficits (15–18). Both during passive and active self-motion, the sensory feedback is thought to be continuously monitored in order to update the self-motion estimate and adjust the internal model if necessary (19,20).

The role of sensory feedback and predictions in self-motion estimation has been studied with closed-loop steering experiments in both monkeys (21–24) and humans (25–28). In these experiments, the self-motion is controlled by a joystick or steering wheel, and the sensory feedback can in principle be predicted based on the steering motor command if an accurate internal model of the steering dynamics is available. Alefantis and colleagues (26) studied human steering behaviour in a virtual environment and found that participants were able to navigate the environment on trials with optic flow cues, but also on interleaved trials without any sensory feedback, suggesting that participants had formed an internal model of the steering dynamics with training. Similarly, Stavropoulos and co-workers (27) studied navigation with optic flow and inertial motion cues while the steering dynamics varied from trial-to-trial according to a random walk (i.e., the dynamics on the previous trial are predictive of the dynamics on the current trial), from responsive to sluggish steering control. Their participants could steer accurately whenever optic flow cues were provided, but less so when only nonvisual, inertial motion cues were available, and the motion had a close to constant velocity. It is thus not evident that inertial motion cues alone can be used to build an internal model by which the generated self-motion can be predicted based on the steering motor command under changing steering dynamics. This is the topic of the present study.

We have previously examined the role of predictions and sensory feedback, in particular inertial motion cues, in self-motion estimation in a steering experiment in which the steering dynamics changed only twice during the experiment (28). Seated on a linear motion sled, participants were instructed to align their body with a memorized visual target using a steering wheel that controlled their lateral body motion. We found that participants responded rapidly (i.e., made within-trial adjustments to their steering movement) to the sudden step changes in the steering dynamics (i.e., the gain between the steering wheel angle and their body velocity). Across trials, participants’ performance gradually improved further by adjusting to the new steering dynamics. One explanation of these findings is that participants built an internal model of the steering dynamics, which transforms the steering motor commands into predicted inertial feedback, that they continued to update throughout the experiment based on the inertial motion cues (19,28). Another explanation is that participants simply relied on path integration mechanisms (29–31), estimating their location relative to the target by integrating the inertial motion cues over time without building an internal model of the steering dynamics. In the present study we aim to distinguish between these two explanations (internal model versus path integration), taking inspiration from studies on the adaptation of reaching movements.

Various studies (32,33) studied visuomotor adaptation of reaching movements while the uncertainty of the visual feedback about the reach endpoint and the uncertainty of the spatial mapping between the reach endpoint and the visual feedback was varied. It was found that adaptation proceeded slower with higher visual feedback uncertainty and faster with higher spatial mapping uncertainty. Gonzalez Castro and colleagues (34) compared adaptation to a force field that varied in strength unpredictably across trials or to a force field that followed a random walk across trials. They found that participants relied more on sensory feedback in the unpredictable condition, while trusting sensory predictions more in the random walk condition. Based on a similar paradigm, it has been proposed that the brain adjusts its sensitivity to errors based on the sequence and environmental context in which those errors occur (35).

In the present study, we used a similar experimental design to dissociate the contribution of inertial feedback and inertial predictions in self-motion estimation during driving. Participants steered a linear sled on which they were seated to translate their body to a memorized visual target. We varied the gain between the steering wheel angle and the velocity of the sled across trials in three different ways: a white noise condition (unpredictable gain), a random walk condition (moderately predictable gain) and a constant gain condition (highly predictable).

We examined the steering behaviour for within-trial responses to the inertial feedback and inertial predictions based on an internal model of the steering dynamics. Furthermore, we introduced large jumps in the gain (i.e., step trials) near the end of each trial block to assess whether differences in steering behaviour across the conditions were directly visible in corrective responses on these trials. If participants simply integrated the inertial motion information over time to estimate the travelled distance (path integration hypothesis), we would expect to see no differences in the steering behaviour across the three conditions. In contrast, if participants did take the across-trial predictability of the gain into account (internal model hypothesis), we would expect the gain from previous trials to have a stronger influence on responses in the random walk condition compared to the white noise condition.

## Methods

### Participants

The study was approved by the ethics committee of the Faculty of Social Sciences of Radboud University Nijmegen, the Netherlands. Twenty-six naïve participants took part in the study (7 men and 19 women; 18-30 years old) and gave their written informed consent before the start of the experiment. They reported to have normal or corrected-to-normal vision, normal hearing, and no history of motion sickness. The experiment took around 90 minutes per participant, and participants were compensated with course credit or €15,00.

### Setup

**Figure 1:**
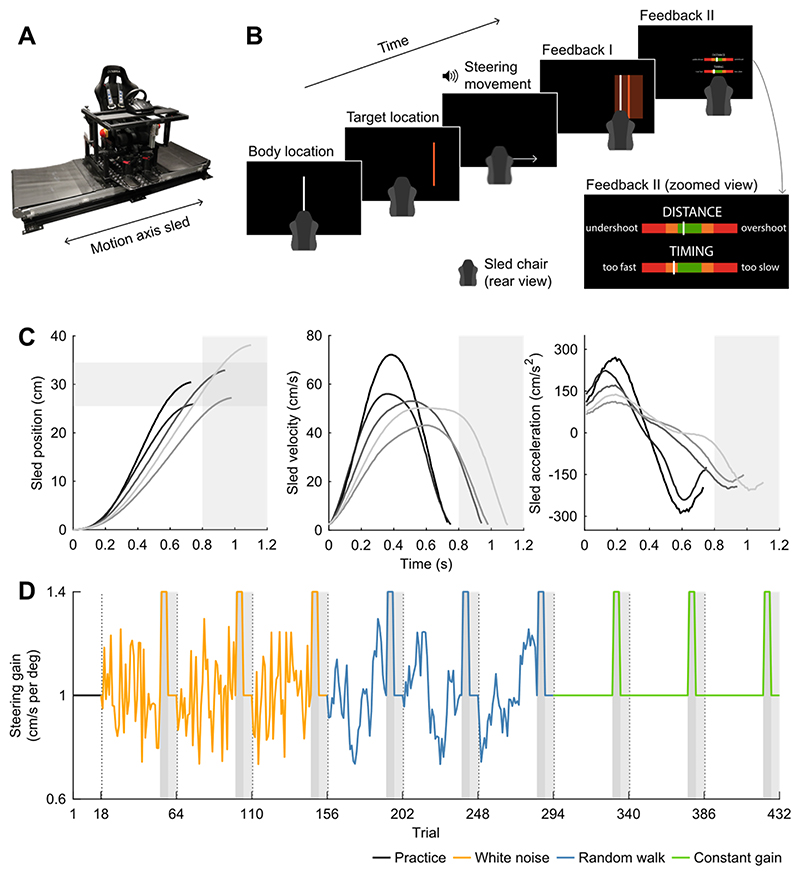
Experimental setup and paradigm. A) Experimental setup. Participants were seated with their interaural axis aligned with the motion axis of the sled and turned a steering wheel to control the sled velocity. B) Experimental paradigm. Participants were first shown their location as a white line, followed by the target location as an orange line. After the disappearance of the target location, a beep instructed participants to turn the steering wheel to translate their body and align it with the memorized target location. When the sled speed was again close to 0 cm/s, visual feedback about the displacement error (Feedback I and II) and the movement duration (Feedback II) was provided. Inset shows the zoomed view of the feedback bars in Feedback II. C) Sled position, velocity and acceleration as a function of time (aligned to movement onset) for five representative condition-specific trials from five different participants in the constant gain condition (grey lines). For each trial, the measured absolute sled position relative to the start location, the absolute sled velocity encoded by the steering wheel angle, and the sled acceleration, computed by low-pass filtering the derivative of the encoded sled velocity using a moving average filter with a window length of nine samples, are shown. D) Example of the steering gain across trials. Each participant completed nine trial blocks. Each trial block started with 36 condition-specific trials, in which the gain varied from trial-to-trial (white noise and random walk condition) or remained the same (constant gain condition). Participants were exposed to the same gains across the white noise and the random walk condition, but trials were organized such that their lag-1 autocorrelation was close to zero in the white noise condition and above 0.8 in the random walk condition. Each trial block was concluded with a baseline trial (gain of 1.0 cm/s per deg), followed by four step trials (high gain of 1.4 cm/s per deg; dark grey areas) and six washout trials (gain of 1.0 cm/s per deg; light grey areas). Participants completed three trial blocks per condition, each followed by a short break (dashed vertical lines), and completed 18 practice trials before the experiment (gain of 1.0 cm/s per deg).

Participants were seated on a custom-built linear motion platform, also called the sled, and used a steering wheel to control the sled speed (Fig. 1A). They sat with their interaural axis aligned with the motion axis of the sled, such that they were laterally translated. They were restrained by a five-point seat belt and could stop the sled motion at any time by pressing one of the emergency buttons on either side of the sled chair. The experiment was performed in darkness. Care was taken to ensure that no visual cues other than the experimental stimuli were present. The sled was powered by a linear motor (TB15N; Tecnotion, Almelo, The Netherlands) and controlled by a servo drive (Kollmorgen S700; Danaher, Washington, DC, USA). The sled track was approximately 93 cm long. Sled position was measured with a linear optical encoder with a resolution of 40 µm (RSF, Tarsdorf, Austria). The steering wheel (G25 Racing Wheel; Logitech, Lausanne, Switzerland) was mounted at a comfortable handling distance in front of the participant at chest level and had a resolution of 0.0549° and a range of rotation from -450° to +450°. The steering wheel angle was recorded at 100 Hz and served as an unfiltered input signal for the control of the speed (not position) of the sled. Using velocity control feels intuitive to the participants, and most like actual driving.

Participants viewed a 55-inch OLED screen (55EA8809-ZC; LG, Seoul, South Korea) with a resolution of 1920 x 1080 pixels and a refresh rate of 60 Hz, positioned centrally in front of the sled track at a viewing distance of approximately 170 cm, and wore noise-cancelling earphones to mask auditory cues induced by the sled motion with white noise sounds (QuietComfort 20; Bose Corporation, Framingham, MA, USA). The experiment was controlled using custom-written software in Python (v.3.6.9; Python Software Foundation).

### Paradigm

Figure 1B shows the order of events during an experimental trial. At the start of a trial, a vertical white line aligned with the body midline (width 0.3 cm and height 25.4 cm) was presented on the screen for 1 second, which represented the start location of the body. After this, a vertical orange line (width 0.3 cm and height 25.4 cm) was presented on the screen for 1 second, representing the target location. The target location was alternately presented to the left and to the right of the start location of the body. The target distance, defined as the distance between the start location of the body and the target location, was always 30 cm. Participants were not informed about the fixed target distance. Of note, because the key conditions had a variable gain, the mapping between the rotation of the steering wheel and the displacement was also variable.

After disappearance of the target, a short beep was played via the earphones to instruct the participant to rotate the steering wheel to align their body midline with the memorized target location. The sled motion started when the participant turned the steering wheel 0.0549 deg (the smallest detectable change) away from the steering wheel angle at trial start. The steering wheel angle at trial start was typically between -20 and 20 deg, with 0 deg representing the centre of the range. The angle of the steering wheel relative to the angle at trial start encoded the velocity of the sled, but the exact steering gain changed throughout the experiment (see below). The latency between the rotation of the steering wheel and the translation of the sled was approximately 20 ms (third quartile of 24 ms). This latency was estimated by computing the difference in time between the time points the absolute velocity encoded by the steering wheel angle and the absolute velocity of the sled, computed based on the measured sled position, exceeded 1 cm/s. The maximum speed of the sled was set to 100 cm/s (which corresponds to 3.6 km/h which is slightly lower than normal walking speed). If the steering wheel angle encoded a higher sled speed, it was capped at this maximum speed. The sled stopped when the steering wheel angle fell within -2 to 2 deg from the start angle, or when the steering wheel angle fell within -6 to 6 deg from the start angle and remained constant for 100 ms or started rising again (stopped steering prematurely or started a new steering movement). If the sled reached one of the ends of the track, it also stopped. Figure 1C shows the sled position, velocity and acceleration as a function of time for five representative trials from five different participants. White noise was played via the earphones during the steering movement to mask auditory cues induced by the sled motion.

After the sled stopped, participants received feedback about their performance. First, both the current location of the body and the target location were presented on the screen for 1 s. This informed participants about how far they ended up from the target location and whether they undershot or overshot the target location. To encourage participants to be as accurate as possible, participants received “hit” feedback if the distance between the current location of the body and the target location was smaller than 4.5 cm. This “hit” area was represented on the screen by a translucent orange rectangular area (width 9 cm and height 25.4 cm) horizontally centred on the target location (Feedback I in Fig. 1B). After this, two horizontal feedback bars (width 15.2 cm and height 1 cm) were shown for 2 s (Feedback II in Fig. 1B). The centre of the feedback bars was green, flanked by orange and red areas towards the edges. A white bar on the upper feedback bar reiterated the displacement error, with the centre of the green area corresponding to the target location, and the left and right edges of the green area corresponding to an undershoot and overshoot of 4.5 cm, respectively (i.e., the “hit” window). A cheerful sound was played via the earphones if the participant “hit” the target. A white bar on the lower feedback bar showed the movement duration. Participants were encouraged to finish their steering movement within 800-1200 ms from movement start to ensure suprathreshold vestibular stimulation while remaining below the maximum sled speed and keep the vestibular range for all participants similar. The centre of the green area of the feedback bar corresponded to a movement duration of 1000 ms, and the left and right edges of the green area corresponded to a movement duration of 800 and 1200 ms, respectively. If the displacement error or the movement duration was out of bounds (i.e., actual location of the white bar was more extreme than the left and right outer edges of the red areas of the feedback bar, corresponding to movement durations of 200 ms and 1800 ms, respectively), the white bar was presented on the outer edge of the feedback bar closest to the true location.

The next trial started after the feedback had disappeared. If the location of the sled at the end of the trial restricted its motion on the next trial to less than 45 cm, the sled was first passively moved to a new starting location. This starting location was 15 cm away from the middle of the sled track in the direction opposite of the upcoming target location, leaving approximately 60 cm for the upcoming displacement.

As described above, the steering gain (i.e., the gain between the angle of the steering wheel and the velocity of the sled) changed throughout the experiment. All participants experienced three different conditions: a random walk condition, a white noise condition, and a constant gain condition (Fig. 1D). In total, the experiment consisted of nine trial blocks, with three trial blocks per condition. Each trial block started with 36 trials specific to the condition. On the last of these condition-specific trials, the steering gain was always 1.0 cm/s per deg (baseline trial), and this trial was always followed by four trials with a high gain of 1.4 cm/s per deg (step trials) and six trials with again of 1.0 cm/s per deg (washout trials).

For the random walk condition, the gains of the other condition-specific trials were generated backwards, starting from the baseline trial with a gain of 1.0 cm/s per deg, in the following way:

(1) $$gain_{i}=\;gain_{i+1}+\;noise\;$$

in which *i* is the trial number. Noise samples were drawn from a Gaussian distribution with a mean of 0 cm/s per deg and a standard deviation of 0.1 cm/s per deg. Random walks were drawn until a walk (excluding the baseline trial) met the following criteria: a mean gain between 0.99 and 1.01 cm/s per deg, a standard deviation between 0.139 and 0.141 cm/s per deg, and a lag-1 autocorrelation value higher than 0.8 (i.e., high predictability). Autocorrelation values were computed by dividing the autocovariance values by the variance of the gains, such that the autocorrelation values fell within -1 to 1. We controlled the standard deviation to ensure spread in the gains while avoiding gains more extreme than the gain on the step trials. The procedure was repeated three times per participant, yielding three random walks per participant.

For the white noise condition, the gains from the three random walks, except for the baseline trials, were shuffled. This was done 10,000 times per walk, and for each walk the instance with the lowest absolute lag-1 autocorrelation value was selected (all between 0.001 and -0.001). This way, the condition-specific trials in the white noise trial blocks had the same means and standard deviations as the condition-specific trials in the random walk trial blocks. In the constant gain condition, all 36 condition-specific trials in the three trial blocks had a gain of 1.0 cm/s per deg.

The conditions were presented in a random order per participant. The number of repetitions of all six possible combinations was balanced across participants whose data were included in the analysis (see below). The three trial blocks per condition were presented consecutively but in random order. At the end of each trial block, the percentage of “hit” trials was presented on the screen, followed by a short break (> 45 seconds) during which the lights in the experimental room were turned on to prevent dark adaptation. Before the experiment, all participants completed 18 practice trials with a gain of 1.0 cm/s per deg, during which the experimenter was present for task instructions. In total, each participant completed 432 trials.

### Data analysis

Data were processed offline in MATLAB (v.R2017a; the MathWorks, Inc., Natick, MA). Data from two participants were excluded from the analysis because of their relatively low scores (average percentage of “hit” trials across trial blocks 48 and 58%; range included participants 66-90%). Trials during which participants rotated the steering wheel less than 7.5 deg or displaced the sled in the direction opposite of the target were excluded from the analysis. Additionally, trials during which the speed encoded by the steering wheel angle reached the set maximum of 100 cm/s or during which the sled reached one of the ends of the sled track were excluded. On average, one trial was excluded per participant (range 0-2 trials).

Movement onset was defined as the first time point that the steering wheel was rotated more than 2 deg. Movement end was taken as the time point the sled stopped, or was corrected offline if the steering wheel angle remained constant for at least 100 ms or reached a local minimum between -7.5 and 7.5 deg (slightly stricter than the window from -6 to 6 deg used during the experiment). Movement duration was defined as the time between movement onset and movement end. Displacement error was defined as the difference between the location of the body at movement end and the target location. Negative errors represent undershoots and positive errors represent overshoots.

To examine whether the predictability of the gains affected the steering behaviour on the condition-specific trials in the white noise and random walk condition, we separated the trials based on the gain on the current trial and the gain on the previous trial. First, the absolute steering wheel angle traces as a function of time were normalized by subtracting the mean absolute steering wheel angle traces across the 36 condition-specific trials within the trial block. Then, for each trial block, we selected the 12 condition-specific trials with the lowest and the 12 condition-specific trials with the highest gain (i.e., current gain), or the subsequent trials (i.e., previous gain), and computed the average across trials within each group. Trials from the constant gain condition were not included in this analysis, because the gain was kept constant throughout the condition-specific trials, making it impossible to split the trials based on the gain.

### Statistics

Statistical analyses were done in MATLAB and R (v.4.0.1; see (36)). The alpha value for statistical significance was set to .05, and this value was Bonferroni-corrected in case of multiple comparisons (exact value of alpha specified with the results of the tests). To compare the overall performance across conditions and trial block repetitions, we examined the average displacement error, movement duration and maximum absolute steering wheel angle across trials within a block using a two-way repeated-measures ANOVA with condition (white noise, random walk, and constant gain) and trial block number (first, second, and third repetition) as within-subject factors using the ez-package in R (v.4.4-0; see (37)). The results were adjusted according to the Greenhouse-Geisser correction in case of violations of sphericity, and we report the generalized eta-squared $\left(\eta_{G}^{2}\right)$ as a measure of the effect size. To compare the steering behaviour on the condition-specific trials with the lowest and the highest gains, as well as the steering behaviour on the subsequent trials, across the white noise and random walk condition, we averaged the normalized steering wheel angle traces of each group across trial blocks within a condition and compared the steering wheel angle traces across the two conditions at each time point with a paired samples t-test in MATLAB. We did this separately for the trials with the lowest and the highest gains (i.e., current gain) and the subsequent trials (i.e., previous gain), and for the low and high gain trial groups. To examine the responses to the step changes in the gain across conditions, we averaged the displacement error, movement duration and maximum absolute steering wheel angle on the baseline trial and the step trials across trial blocks within a condition and examined differences between the trials and conditions using a two-way repeated-measures ANOVA with condition (white noise, random walk, and constant gain) and trial (baseline and first, second, third, and fourth step trial) as within-subject factors. We used paired-samples t-tests to directly compare the groups post hoc.

## Results

We used a closed-loop steering experiment in which participants steered a linear sled to align their body with a memorized visual target. We varied the steering gain in three different ways and examined whether participants took the predictability of the gain into account in their steering behaviour (internal model hypothesis) or whether participants simply integrated the inertial motion cues over time (path integration hypothesis).

### General observations

**Figure 2:**
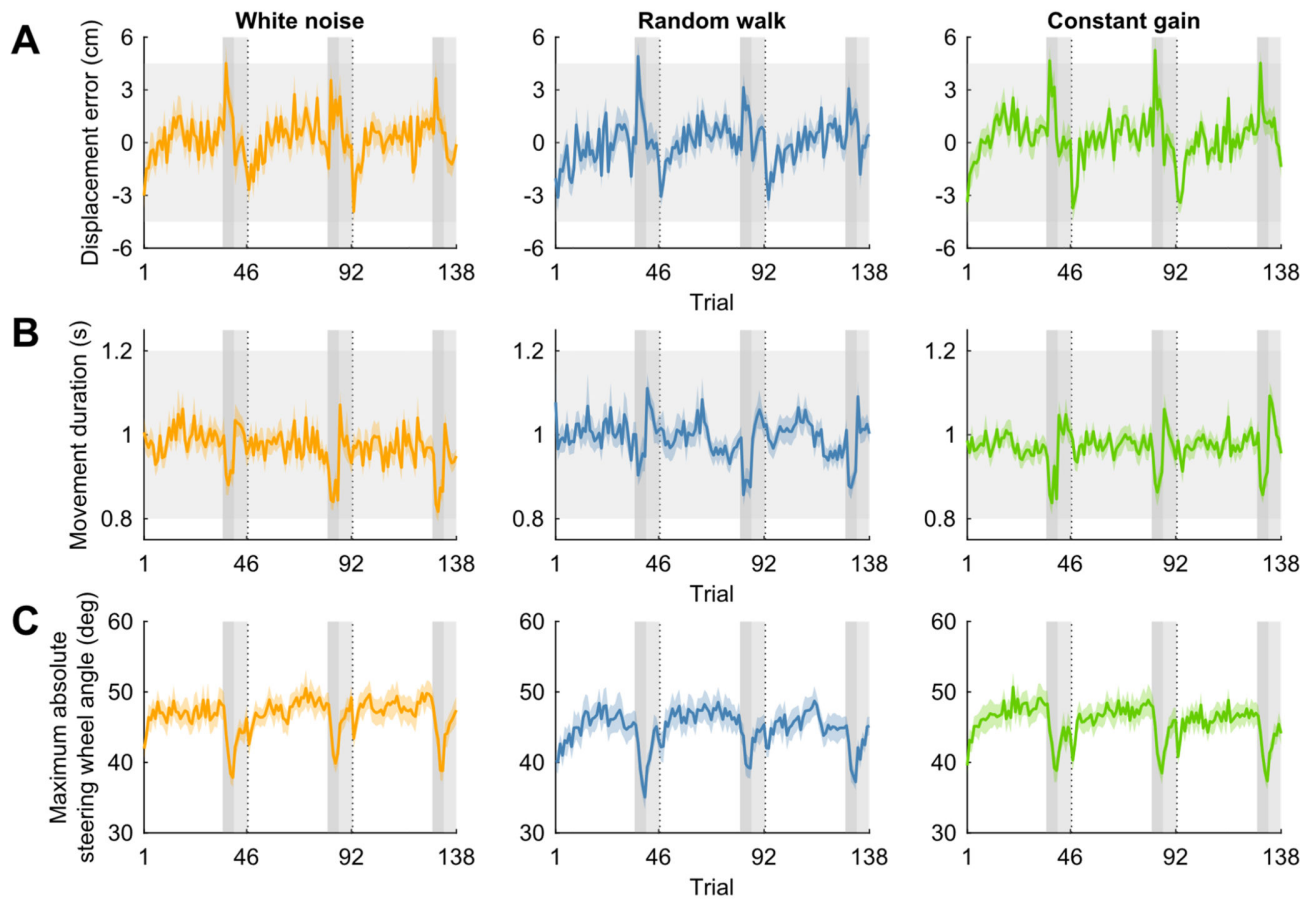
Displacement error, movement duration and maximum absolute steering wheel angle. A) Mean displacement error across participants as a function of trial number grouped based on the experimental condition (panels). Negative numbers represent undershoots; positive numbers represent overshoots. Coloured shaded areas represent between-subjects means ± SE. Participants completed three trial blocks per condition in sequence and the conditions were presented in a random order per participant. Each trial block was concluded with a baseline trial, followed by four step trials with a high gain of 1.4 cm/s per deg (dark grey vertical areas) and six washout trials with a gain of 1.0 cm/s per deg (light grey vertical areas). Dashed vertical lines represent breaks and horizontal light grey bands show the range of displacement errors within which participants “hit” the target. B) Same configuration as in A, but with the mean movement duration across participants. Horizontal light grey bands show the time window within which participants were encouraged to finish their steering movement. C) Same configuration as in A, but with the mean maximum absolute steering wheel angle across participants. N = 24 participants.

Figure 2A shows the average displacement error across participants as a function of trial number for each of the three conditions. Participants hit the target on average in 75% of trials (range 66-90%). The average displacement error was close to zero in all three conditions (white noise: *M* = 0.25 cm, *SD* = 1.12 cm; random walk: *M* = 0.03 cm, *SD* = 1.06 cm; constant gain: *M* = 0.30 cm, *SD* = 1.12 cm) and in all three trial blocks within a condition (first repetition: *M* = 0.18 cm, *SD* = 1.22 cm; second repetition: *M* = 0.32 cm, *SD* = 1.05 cm; third repetition: *M* = 0.09 cm, *SD* = 1.02 cm). In line, the overall displacement error did not differ significantly across the conditions $(F_{2,46}=1.23,p=.301,\eta_{G}^{2}\;=.011\;)\;$ or across the three trial blocks within a condition $\left(F_{2,46}=1.58,p=.218,\eta_{G}^{2}=.008\right)$. Additionally, there was no significant interaction effect between the condition and the trial block number on the displacement error $\left(F_{4,92}=1.02,p=.401,\eta_{G}^{2}=.011\right)$. The included participants were thus able to hit the target in most trials.

Figure 2B shows the average movement duration in the same format as in Figure 2A. On average, participants finished their steering movement within the imposed time window from 800 to 1200 ms in all three conditions (white noise: *M* = 970 ms, *SD* = 97 ms; random walk: *M* = 994 ms, *SD* = 108 ms; constant gain: *M* = 972 ms, *SD* = 86 ms) and in all three trial blocks (first repetition: *M* = 987 ms, *SD* = 101 ms; second repetition: *M* = 974 ms, *SD* = 99 ms; third repetition: *M* = 975 ms, *SD* = 93 ms). There was no significant difference in the overall movement duration across conditions $(F_{1.61,36.95}=\;1.78,p=.188,\eta_{G}^{2}=.012)$ or trial blocks $\left(F_{2,46}=1.32,p=.277,\eta_{G}^{2}=.004\right)$. Additionally, there was no significant interaction effect between the condition and the trial block number on the movement duration $\left(F_{4,92}=1.32,p=.268,\eta_{G}^{2}=.004\right)$.

Figure 2C shows the average maximum absolute steering wheel angle in the same format as in Figure 2A. The average maximum absolute steering wheel angle was similar across conditions (white noise: *M* = 46.7 deg, *SD* = 7.0 deg; random walk: *M* = 44.9 deg, *SD* = 6.9 deg; constant gain: *M* = 45.7 deg, *SD* = 7.0 deg) and repetitions (first repetition: *M* = 45.4 deg, *SD* = 7.1 deg; second repetition: *M* = 46.2 deg, *SD* = 7.0 deg; third repetition: *M* = 45.7 deg, *SD* = 6.9 deg). In line, the maximum absolute steering wheel angle was not significantly affected by the condition $\left(F_{2,46}=2.13,p=.130,\eta_{G}^{2}=.011\right)$ or the trial block number $\left(F_{1.48,33.96}=1.23,p=.294,\eta_{G}^{2}=.003\right)$, nor was there an interaction effect $\left(F_{2.93,67.38}=1.14,p=.338,\eta_{G}^{2}=.002\right)$.

### Condition-specific trial analysis

The above findings suggest that, overall, the steering behaviour was similar across conditions and trial blocks. However, in the white noise and random walk condition, the steering gain varied from trial-to-trial and across participants. Averaging across trials within a trial block and across participants could therefore mask effects of the gain on the steering behaviour at the single-trial level.

We therefore examined how deviations from the mean steering response relate to the gain of the current trial, separately for each of the two conditions. For each trial block, we identified the 12 trials with the highest steering gain and the 12 trials with the lowest steering gain. If participants can anticipate the gain from the one trial to the next, as is possible in the random walk condition, we would expect to see a positive deviation already from the start of the trial in the low-gain trials, and a negative deviation at the start of the high-gain trials. In contrast, when the steering gain is unpredictable, as in the white noise condition, such deviations should only emerge later in the trial, once participants have had the opportunity to detect and respond to the actual gain.

**Figure 3:**
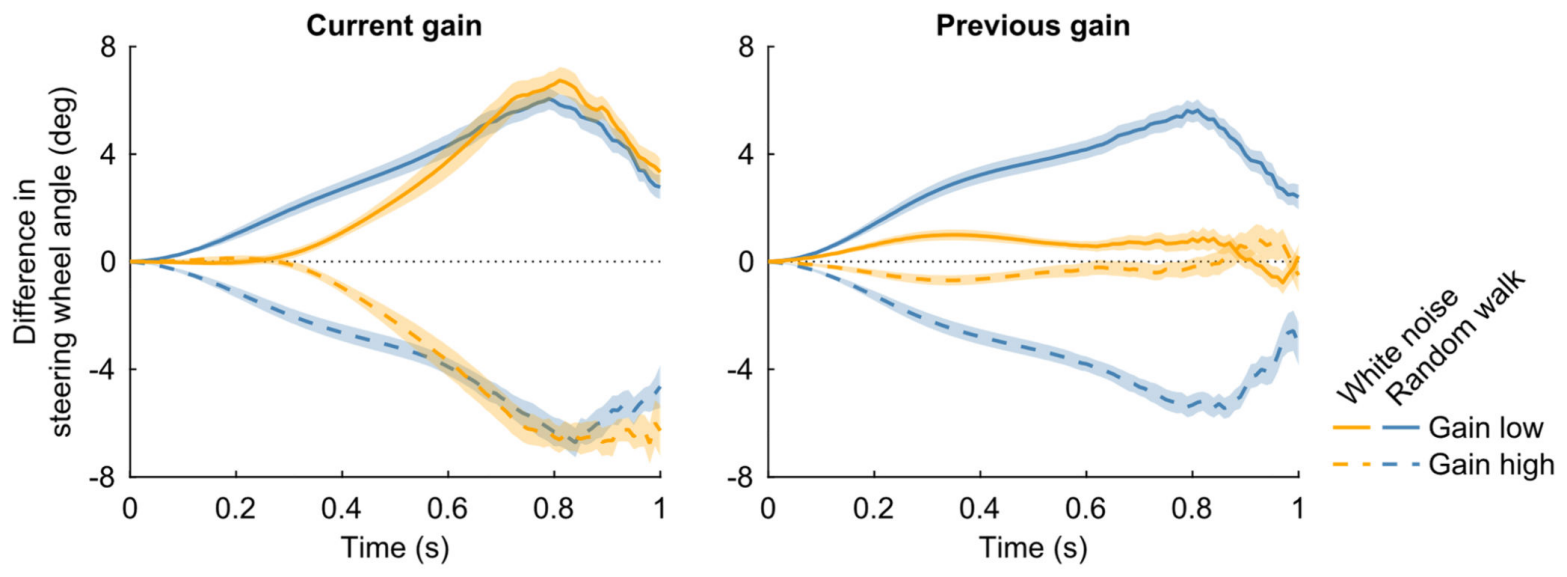
Steering behaviour on the condition-specific trials separated based on the current and the previous gain. For each trial block, the 12 condition-specific trials with the lowest gain and the 12 condition-specific trials with the highest gain were selected. The absolute steering wheel angle traces were normalized by subtracting the mean absolute steering wheel angle traces across the 36 condition-specific trials per trial block, and the average was computed across trials within each group, across trial blocks within each condition, and across participants. Results are shown for the trials with the lowest and the highest gains (left panel) as well as the subsequent trials (right panel) and are grouped based on experimental condition (coloured lines) and gain (solid and dashed lines). Negative numbers represent steering wheel angles smaller than the mean; positive numbers represent steering wheel angles larger than the mean. Coloured shaded areas represent between-subjects means ± SE. N = 24 participants.

Figure 3, left panel, demonstrates a significant difference in steering responses between the two conditions. In the white noise condition, the steering wheel angle followed the mean steering wheel angle during approximately the first 300 ms of the trial, both on trials with a low and a high gain. After this, the steering wheel angle increased on trials with a low gain and decreased on trials with a high gain. Participants thus reacted adequately to changes in the gain by steering against the gain change. In contrast, in the random walk condition, the steering wheel angle was higher than the mean steering wheel angle on trials with a low gain already from the start of the trial, and lower on trials with a high gain. The steering wheel angle traces differed significantly between the white noise condition and the random walk condition for trials with a low gain (significant from 140 to 500 ms after movement onset; range p-values from p = .00048 to p < .0001; Bonferroni-corrected α = .00049, based on 101 comparisons across time points) and trials with a high gain (significant from 110 to 430 ms after movement onset; range p-values from p = .00033 to p < .0001; Bonferroni-corrected α = .00049, based on 101 comparisons across time points).

The right panel of Figure 3 presents the same analysis, but with trials grouped according to the previous gain. In the random walk condition, participants clearly adjusted their steering based on the gain from the preceding trial. In contrast, in the white noise condition, the influence of previous gain is minimal, indicating that participants’ steering responses remain close to the mean. This is confirmed by a significant difference in steering wheel angle traces between the white noise condition and the random walk condition for trials with a low gain (significant from 160 to 990 ms after movement onset; range p-values from p = .00049 to p < .0001; Bonferroni-corrected α = .00049, based on 101 comparisons across time points) and trials with a high gain (significant from 200 to 970 ms after movement onset; range p-values from p = .00048 to p < .0001; Bonferroni-corrected α = .00049, based on 101 comparisons across time points). These findings are in line with the internal model hypothesis, as it is more advantageous to take the previous gain into account in the random walk condition because it is more predictive of the current gain due to the high autocorrelation.

### Step trial analysis

To examine whether these differences in the steering response across the conditions were also directly visible in the steering behaviour after larger jumps in the gain, we added four step trials with a high gain of 1.4 cm/s per deg to the end of each trial block. All step trials were preceded by a baseline trial and were followed by six washout trials, all with a gain of 1.0 cm/s per deg.

**Figure 4:**
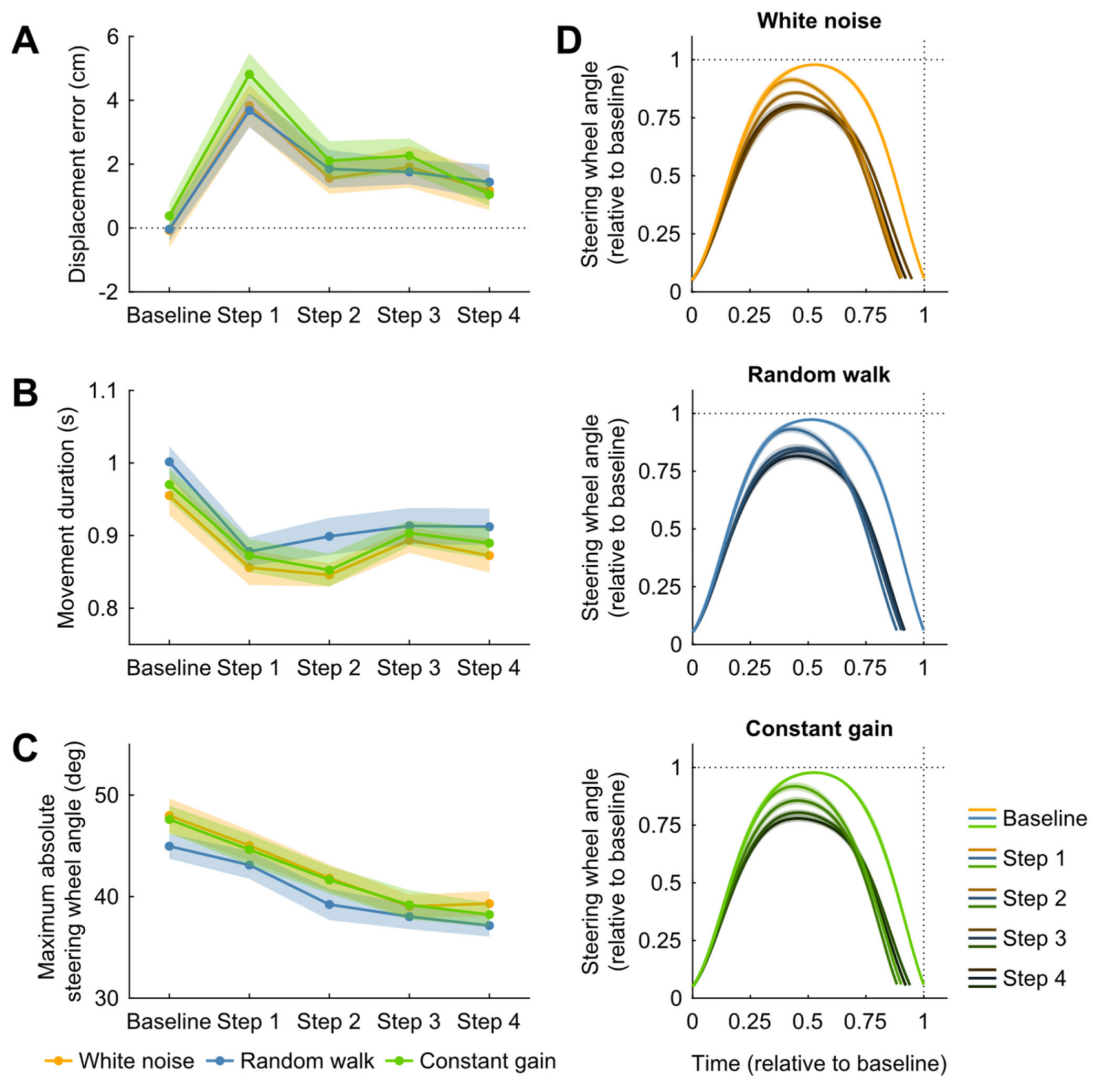
Steering behaviour on baseline and step trials. A) Mean displacement error across trial blocks and participants as a function of the trial grouped based on the experimental condition (coloured lines). Negative numbers represent undershoots; positive numbers represent overshoots. Coloured shaded areas represent between-subjects means ± SE. B) Same configuration as in A, but with the mean movement duration across trial blocks and participants. C) Same configuration as in A, but with the mean maximum absolute steering wheel angle across trial blocks and participants. D) Average absolute steering wheel angle as a function of time across trial blocks and participants for the baseline and step trials grouped based on the experimental condition (vertical panels). Values were normalized relative to baseline. N = 24 participants.

Figure 4 shows the steering behaviour for the baseline and step trials, grouped based on the condition and averaged across trial blocks and participants. To examine participants’ responses to the step changes in the gain, we compared the baseline trial and the step trials across conditions. In all conditions, the average displacement error on the first step trial was positive and larger than the average displacement error on the baseline trial (Fig. 4A). There was a significant main effect of the trial on the displacement error $\left(F_{4,92}=18.27,p<.001,\eta_{G}^{2}=.205\right)$, and post hoc paired-samples t-tests revealed that there was a significant difference between the baseline trial and all four step trials (range p-values from *p* = .002 to *p* < .0001; Bonferroni-corrected *α* = .005), and between the first step trial and the subsequent step trials (all p-values < .0001; Bonferroni-corrected *α* = .005). There was no significant main effect of the condition on the displacement error $\left(F_{2,46}=0.83,p=.444,\eta_{G}^{2}=.006\right)$, or a significant interaction effect $\left(F_{8,184}=0.41,p=.912,\eta_{G}^{2}=.007\right)$. However, in all three conditions the overshoot of the target location on the first step trial was smaller than 12 cm, which is the displacement error that would be expected if participants did not respond to the increase in the gain (target distance of 30 cm and gain increase from 1.0 cm/s per deg to 1.4 cm/s per deg). This suggests that participants changed their steering movement online during the first step trial to compensate for the increase in the gain in all three conditions.

This is confirmed by changes in the movement duration (Fig. 4B) and maximum absolute steering wheel angle (Fig. 4C) from the baseline trial to the step trials. The movement duration differed significantly across trials in all conditions $\left(F_{4,92}=30.57,p<.001,\eta_{G}^{2}=.122\right)$, with significantly shorter movement durations on the step trials than on the baseline trial (all p-values < .0001; Bonferroni-corrected *α* = .005). Interestingly, the movement duration increased again across the step trials, with significantly longer movement durations on the third step trial than on the first and second step trials (range p-values from *p* = .0001 to *p* < .0001; Bonferroni-corrected *α* = .005). There was no significant main effect of the condition on the movement duration $\left(F_{2,46}=1.89,p=.162,\eta_{G}^{2}=.019\right)$, or an interaction effect $\left(F_{8,184}=0.75,p=.644,\eta_{G}^{2}=.004\right)$. Similarly, the maximum absolute steering wheel angle differed significantly across trials in all conditions $\left(F_{4,92}=92.17,p<.001,\eta_{G}^{2}=.205\right)$, with a significantly larger maximum absolute steering wheel angle on the baseline trial than on all four step trials (all p-values < .001; Bonferroni-corrected *α* = .005). The maximum absolute steering wheel angle continued to decrease significantly across the first three step trials (all p-values < .001; Bonferroni-corrected *α* = .005). There was no significant main effect of the condition on the maximum absolute steering wheel angle $\left(F_{2,46}=3.01,p=.059,\eta_{G}^{2}=.020\right)$, or an interaction effect between the trial and the condition $\left(F_{8,184}=0.79,p=.611,\eta_{G}^{2}=.003\right)$. Participants seemed to fine-tune their steering behaviour after the large jump in the gain by increasing the movement duration again slightly and continuing to decrease the maximum absolute steering wheel angle across the step trials, thereby minimizing the displacement error while simultaneously adhering to the imposed time window.

The similarity in the correction across conditions and the fine-tuning of the steering behaviour across the step trials is also shown in Figure 4D. In this figure, the steering wheel angle as a function of time is normalized relative to the baseline trials. For each participant and trial block, we first resampled the steering wheel angle traces of the baseline and the four step trials to 200 samples per trial using linear interpolation. The movement duration and steering wheel angles on these trials were then normalized by dividing them by the movement duration and the maximum absolute steering wheel angle of the corresponding baseline trial, respectively. Normalized steering wheel angle traces were averaged across trial blocks and participants, and a corresponding linearly spaced time vector of 200 samples was created for each trial running from zero, representing movement onset, to the mean normalized movement duration across trial blocks and participants.

Figure 4D is included to give a qualitative insight in how the steering develops in the step trials. As shown, participants decreased both the movement duration and the maximum absolute steering wheel angle in response to the increase in the gain from the baseline to the first step trial. They did this already early on within the first step trial. The responses to the higher gain are similar across conditions, but the decrease in the maximum absolute steering wheel angle from the baseline trial to the first step trial seems to be slightly smaller in the random walk condition, as also shown in Figure 4C. Participants continued to decrease the maximum absolute steering wheel angle across the subsequent step trials, while slightly increasing the movement duration again towards the baseline movement duration.

## Discussion

In this study, participants used a steering wheel to move their body to a memorized visual target location. They were exposed to three experimental conditions, in which the gain between the steering wheel angle and the velocity of the linear motion platform varied with different levels of predictability from one trial to the next. In the white noise condition, the steering gain varied randomly from trial-to-trial (i.e., not predictable), in the random walk condition it was moderately predictable, and in the constant gain condition it remained constant across trials (i.e., highly predictable). The goal was to examine whether participants took the predictability of changes in the gain into account in their steering behaviour, by forming and relying on an internal model of the steering dynamics, or whether they simply relied on the inertial motion cues in their steering, as in path integration.

By separating the trials based on the gain on the current and the previous trial, we have shown that participants used a different steering response for the white noise and random walk conditions (Fig. 3). In the white noise condition, both the average steering wheel angle across trials with the lowest gains and the average steering wheel angle across trials with the highest gains were similar to the overall average steering wheel angle for approximately the first 300 ms of the steering movement, irrespective of the gain. When we separated the trials based on the gain in the previous trial, it was revealed that participants do only minimally rely on the gain on the previous trial in their steering in the white noise condition. In the random walk condition, the steering behaviour differed between the trials with the lowest gains and the trials with the highest gains already from the start of the trial. When we separated the trials based on the gain on the previous trial, we found similar results. Significant differences across conditions in the effect of the gain on the previous trial showed that participants based their steering behaviour on the gain on the previous trial more in the random walk condition than in the white noise condition. This is a useful strategy given the high autocorrelation in the gains in the random walk condition. We conclude that participants formed an internal representation of the steering dynamics, which is in line with the internal model hypothesis.

In all conditions, including the constant gain condition, participants decreased the maximum absolute steering wheel angle and the movement duration on the first step trial. Across the subsequent step trials, participants improved their adaptation to the new steering dynamics by simultaneously increasing the movement duration and decreasing the maximum absolute steering wheel angle to adhere to the time window imposed in the experiment. These tactful changes in the steering behaviour underline the idea that participants built and updated an internal model of the steering dynamics and the associated self-motion based on the inertial feedback.

It is known that performance feedback can be important in learning an internal model (38), although online feedback during the movement provides more information, enabling, for example, estimation of both body and environmental state (39). Indeed, participants have most likely used performance feedback to update their beliefs and use these in the next trial. However, the performance feedback was of no use for the within-trial compensation (as it came after the trial), which to us provides strong evidence that the internal model of the steering dynamics is primarily derived from inertial motion cues.

In our previous study (28), participants performed a similar steering experiment but the steering gain changed only twice during the whole experiment, comparable with the constant gain condition in the current experiment. We found that participants responded rapidly to these changes in the steering dynamics, suggesting that participants had some expectations about their velocity and the steering dynamics, but we could not further distinguish between the internal model hypothesis and the path integration hypothesis. Here, we dissociate the contribution of predictions and inertial feedback by changing the steering dynamics across trials with different levels of predictability. We show that participants use the inertial feedback during the trial to estimate their self-motion, but also that their steering behaviour depends on the predictability of the steering gain. Important to note, this conclusion is based mainly on the results of an analysis in which we separated the condition-specific trials based on the gain on the current and the previous trial, in which the data from the constant gain condition could not be included. Due to the high predictability of the steering gain, we expected participants to respond slowest on the step trials in the constant gain condition, but the data did not support this notion. To study steering behaviour with a high predictability of the steering gain, future studies could include white noise and random walk conditions with varying levels of variability, similar to Burge and colleagues (32) who studied the trade-off between prediction and estimation based on sensory feedback in reach adaptation.

Stavropoulos et al. (27) also used a closed-loop steering experiment to study the role of inertial feedback and predictions in self-motion estimation. Participants used a joystick to navigate to a target while the steering dynamics changed from trial-to-trial following a bounded random walk. They found that the steering behaviour was biased with responsive steering control without sustained acceleration and concluded that participants were not able to accurately steer and build an internal model of the steering dynamics in such a situation based on the inertial motion cues alone. Our previous results suggested that participants can accurately estimate their self-motion and suggest that they build an internal model of the steering dynamics based on just inertial feedback (28), and here we show that they can even do this under steering dynamics that change from trial-to-trial. This discrepancy between the results might be explained by the fact that the velocity of the motion platform used by Stavropoulos et al. was close to constant. This may have made it more difficult for participants to estimate their self-motion, as the vestibular organs and more specifically the otoliths, which process information about translational motion, are known to be mainly sensitive to acceleration (40–42). Also, participants in our experiments received feedback about their performance at the end of each trial, which is likely to have minimized any possible biases in participants’ self-motion estimates.

The present study is based on previous studies of visuomotor and force field adaptation in reaching movements that examined the role of sensory feedback and predictions (32–34). These studies showed that participants respond faster to perturbations if the mapping between the reaching movement and the sensory feedback is more uncertain. In other words, when the mapping changes frequently, participants update their mapping estimates more quickly in response to errors, as previous estimates are less likely to remain valid. Similar to the present study, Gonzalez Castro et al. (34) examined motor adaptation when the force field perturbation strength varied randomly across trials and when it varied according to a random walk. They showed that participants relied more on predictions of the perturbation in the random walk condition. This is in line with our finding that the effect of the previous gain on the steering behaviour is more pronounced in the random walk condition than in the white noise condition.

Our results suggest that participants build an internal model of the steering dynamics to estimate their self-motion during active steering. We note that the present paradigm cannot distinguish between the type of sensory systems that provide the inertial motion cues. During inertial motion, in addition to the vestibular cues, there are typically also extra-vestibular cues available such as somatosensation (e.g. proprioception and pressure on the body), and sound or noise cues. These extra-vestibular sensory systems cannot be switched off in healthy participants. However, we minimized their effects as participants moved with their interaural axis aligned with the motion axis of the sled and wore noise cancelling headphones during the experiment. Furthermore, both behavioural and electrophysiological studies have shown that self-motion estimation strongly depends on intact vestibular labyrinths (43), which may suggest that the changes of the internal model of the steering dynamics were also primarily driven by vestibular signals.

Multiple studies have looked for neural markers of such internal model of steering and have tried to unravel its location in the vestibular processing pathway (21–24,44). In general, the cerebellum is thought to play an important role in the internal model computations for self-motion estimation (8,19,45,46), also because of its projections to the vestibular nuclei. Neurons in the vestibular nuclei are known to distinguish between active and passive self-motion, being less sensitive to actively generated, and thus predictable, self-motion (47). However, these neurons respond similarly to passive self-motion and self-motion generated by a steering movement (21). One explanation for this may be that the monkeys in the experiment were not trained enough to build an internal model of the steering dynamics. Another explanation may be that the cerebellum does not predict the sensory consequences of the steering movement, and that the internal model of the steering movement is located more downstream in the vestibular processing pathway (26). Similarly, during the processing of the visual reafference of steering movements in monkeys, markers for an internal model were found in the medial superior temporal area (22) and the posterior parietal cortex (44).

The sensorimotor processes that underlie driving have gained additional interest with the development of automated vehicles (48,49). Nash and Cole (49) have described these sensorimotor processes in detail, and have shown that a driver model that includes an internal model of the mapping between the steering wheel angle and the sensory feedback accurately describes human steering behaviour in their experimental set up. Our results are in line with these findings. Based on the predictions of such an internal model, feedforward control actions can be made, which can be extremely important in driving given the delays in the sensorimotor system (48). Along these lines, the present results may also stimulate novel concepts for artificial navigation systems, e.g., those providing independent mobility to sensory-deprived people and vehicle control.

Finally, throughout this paper, we have interpreted our findings as evidence that participants construct an internal model of the steering dynamics. However, it is important to clarify that our results do not allow us to definitively distinguish whether participants are forming and updating an internal prediction of the dynamics themselves, or rather their estimate of the consistency of those dynamics. It is plausible that initial familiarization with the task enables participants to establish a basic internal model of the steering dynamics. During subsequent exposure to the white noise or random walk perturbations, participants may then focus primarily on updating the consistency or reliability of these dynamics, rather than relearning or updating the underlying dynamics themselves. This perspective would be consistent with the recent framework proposed by Heald et al. (50), which differentiates between the formation of a memory (the initial learning of the dynamics) and the updating of that memory (learning about the consistency or reliability of those dynamics).

In conclusion, our results show that participants take the predictability of changes in the steering dynamics into account during driving. This suggests that participants build an internal model of the gain between the steering wheel angle and their self-motion and use this model to predict the inertial reafference in driving and self-motion estimation.

## Figures and Tables

**Figure:**
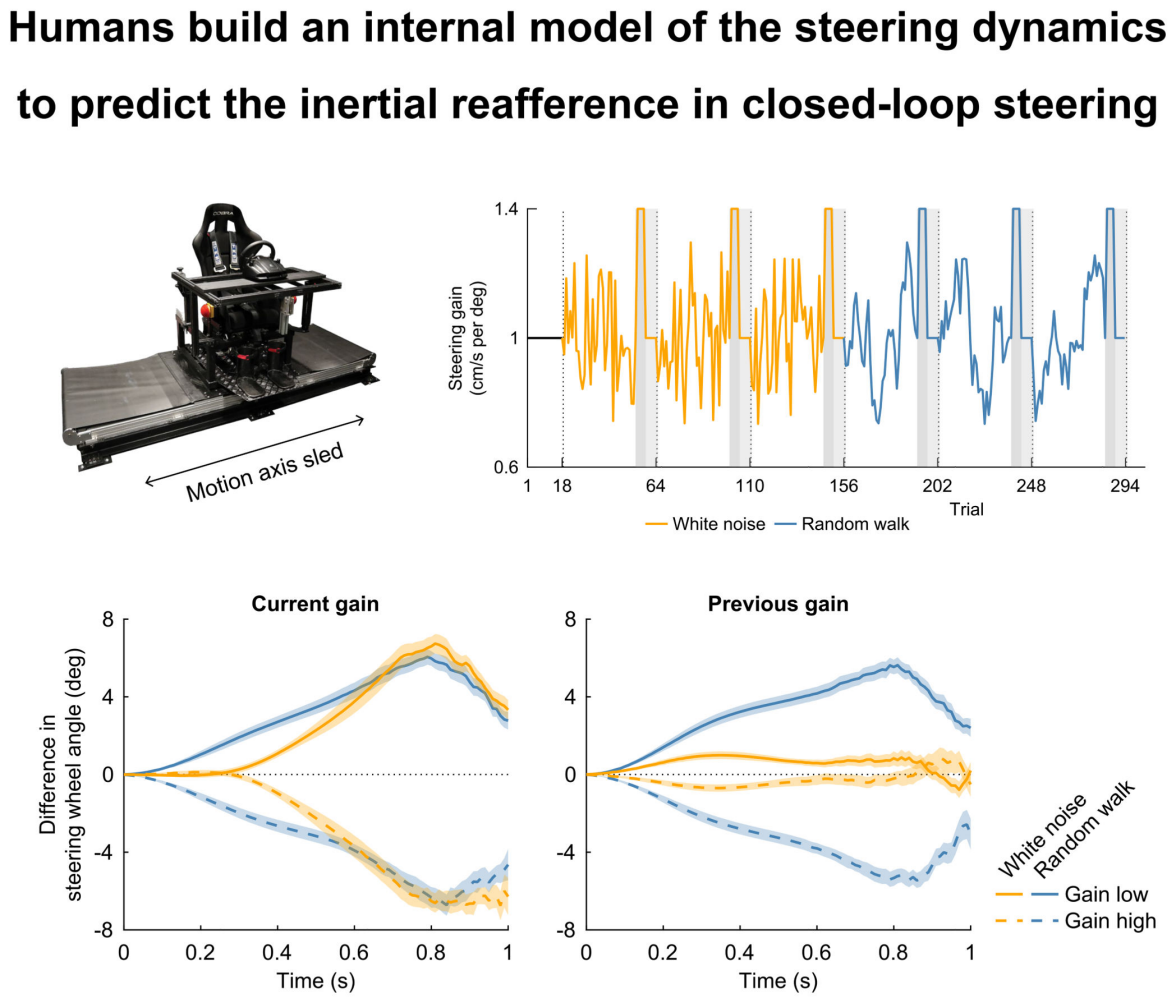


## Data Availability

For review purposes, all data and code are available from the Radboud Data Repository via the following URL: https://data.ru.nl/login/reviewer-2801061450/HA7ROZXPLIGY2QMQZSSIVBMLRIT6GWRFMM5M6TQ. Upon publication, all data and code will be made publicly available via the persistent identifier currently reserved for this collection: https://doi.org/10.34973/szbh-kc35.

## New and newsworthy

We used a closed-loop steering experiment to investigate whether an accurate internal model of the steering dynamics can be learned based on inertial sensory cues. Participants were shown to benefit from partially predictable steering dynamics; they took the across-trial predictability of the steering gain into account to control their steering. This suggests that they can build an internal model to anticipate the inertial reafference in driving and self-motion estimation.
